# Aggressive Recurrence of Primary Hepatic Epithelioid Haemangioendothelioma after Liver Transplantation

**DOI:** 10.1155/2016/6135297

**Published:** 2016-05-18

**Authors:** Qusay A. Abdoh, Asma M. Alnajjar, Faisal A. Abaalkhail, Mohammed Al Sebayel, Hussa F. Al-Hussaini, Waleed K. Al-Hamoudi, Hazem Helmy, Mohamad Almansour, Hussien A. Elsiesy

**Affiliations:** ^1^An-Najah National University, Nablus, State of Palestine; ^2^College of Medicine, Alfaisal University, Riyadh 11533, Saudi Arabia; ^3^Department of Liver and Small Bowel Transplantation and Hepatobiliary and Pancreatic Surgery, King Faisal Specialist Hospital and Research Center (KFSH&RC), P.O. Box 3354, Riyadh 11211, Saudi Arabia; ^4^Department of Pathology and Laboratory Medicine, King Faisal Specialist Hospital and Research Center (KFSH&RC), P.O. Box 3354, Riyadh 11211, Saudi Arabia; ^5^Gastroenterology and Hepatology Unit, Department of Medicine, King Saud University, P.O. Box 2925, Riyadh 11461, Saudi Arabia; ^6^Faculty of Medicine, Minia University, Minia 11432, Egypt; ^7^Critical Care, King Faisal Specialist Hospital and Research Center (KFSH&RC), P.O. Box 3354, Riyadh 11211, Saudi Arabia

## Abstract

HEHE is a rare neoplasm of vascular origin that occurs in the liver; UNOS reported a favorable outcome after liver transplantation in 110 patients with 1-year and 5-year survival of 80% and 64%.* Case Report.* A 40-year-old lady presented with a three-month history of right upper abdominal pain with nausea, vomiting, and significant loss of weight associated with scleral icterus and progressive abdominal distension. Examination revealed jaundice, hepatomegaly, and ascites. Serum bilirubin was 26.5 mg/dL and ALP was 552 CT. Abdomen and pelvis showed diffuse infiltrative neoplastic process of the liver with a mass effect and stretching of the hepatic and portal veins, in addition to bile duct dilatation. Viral hepatitis markers were negative and serum alpha fetoprotein was within reference range. Liver biopsy was consistent with HEHE, with positive endothelial markers (CD31, CD34, and factor VIII-related antigen). She underwent living related liver transplantation on June 2013 and was discharged after 20 days with normal liver enzymes. Four months later, she presented with diffuse disease recurrence. Liver biopsy confirmed disease recurrence; she received supportive treatment and unfortunately she died 2 weeks later.* Conclusion.* HEHE can have rapid and aggressive recurrence after liver transplantation.

## 1. Case Presentation

A 40-year-old female with history of hypertension presented on April 2013 with three-month history of right upper abdominal pain, nausea, vomiting, jaundice, 6 kg weight loss, and progressive abdominal distension. Physical examination revealed jaundice, hepatomegaly, and hard liver with moderate ascites.

Laboratory investigations revealed normal PT/PTT and abnormal liver function: total bilirubin (26.5 mg/dL); direct bilirubin (22.2 mg/dL); AST (369 IU/L); ALT (49 IU/L); alkaline phosphatase (552 IU/L). Viral markers for hepatitis A, B, and C were nonreactive.

Serum alpha fetoprotein was 2.6 *μ*g/L.

Computed tomography (CT) of the chest, abdomen, and pelvis showed diffuse neoplastic infiltrative involvement of the entire liver with sparing of small patchy areas of the liver with a mass effect and no evidence of metastasis. PET scan and bone scan were negative.

US guided liver biopsy was consistent with hepatic epithelioid haemangioendothelioma (HEHE). All tumor cells were positive for endothelial markers such as CD31, CD34, and factor VIII-related antigen. The patient underwent living related liver transplantation on June 2013.

**Figure 1:**
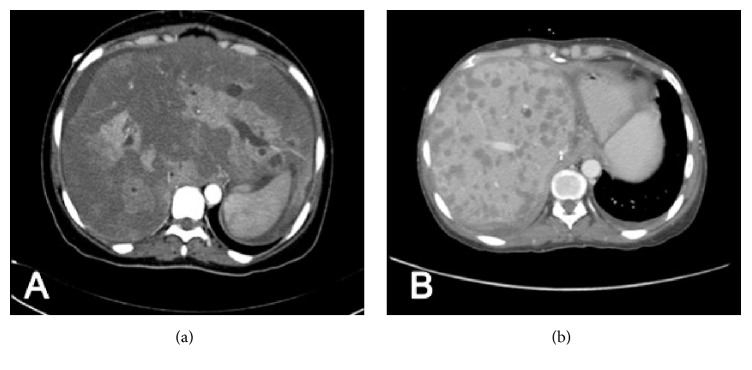
(a) Abdomen CT showing diffuse neoplastic infiltrative involvement of the entire liver with sparing of small patchy areas of the liver. (b) CT of the abdomen showing post-liver transplant recurrence with innumerable diffuse hypodense lesions in the liver.

The explanted liver weighed 3222 grams and measured 33 × 24 × 12 cm. The capsular surface showed foci of capsular retraction (Figure 1(a)). Cross sections of the resected liver showed multiple white geographical areas alternating with the liver tissue, involving the whole surface area. The white areas were rimmed by hyperemic edge (Figure 1(b)).

**Figure 2:**
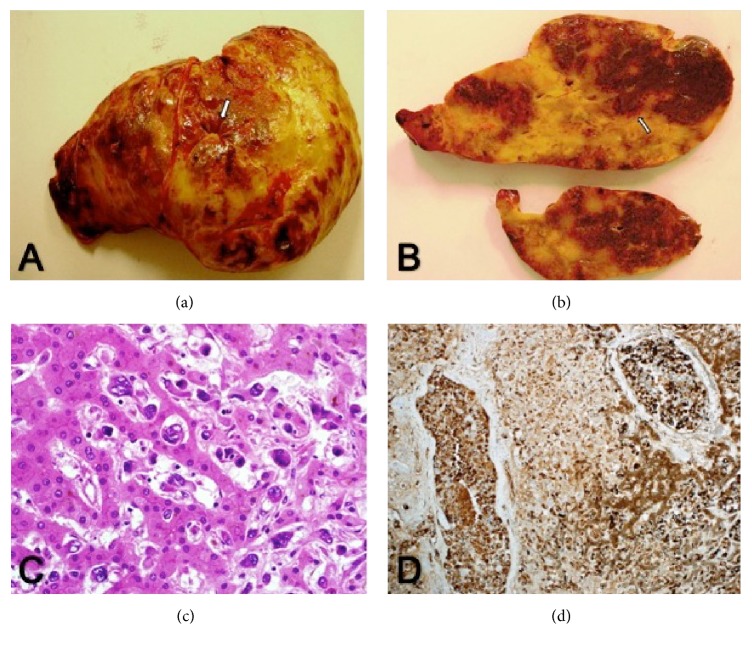
(a) Hepatectomy specimen with focal capsular retraction (white arrow), (b) cross section of the liver showing white sclerotic areas with hyperemic rim (white arrow), (c) malignant epithelioid haemangioendothelioma cells with sinusoidal infiltration (HE ×40), and (d) tumor cells positive for CD31 and CD34.

Microscopic examination revealed diffuse infiltration by malignant epithelioid cells with signet ring morphology and massive areas of necrosis; the cells showed similar positive reaction to endothelial markers (Figures 2(a)–2(d)). The tumor showed aggressive morphology with abundant vascular and neural infiltration. The gall bladder was extensively infiltrated by the same tumor cells, which reached up to the mucosal surface.

Postoperatively, her liver enzymes were normalizing and repeated liver Doppler US after transplantation showed patent vessels and small hepatic hematoma, and she was then discharged on prednisone, tacrolimus, and cellcept.

One month later, she presented with severe abdominal pain and ascites and elevated liver enzymes. CT of the abdomen showed innumerable diffuse hypodense lesions in the liver, enlarged periaortic, periportal, and aortocaval lymph nodes, and moderate ascites. Liver biopsy revealed recurrence of the disease. Patient received supportive treatment, and unfortunately, she passed away a month later.

## 2. Discussion

The first series of 32 HEHE was described in 1984 [1]. Mehrabi et al. reviewed 434 reported cases between 1984 and 2005 in MEDLINE; 44% of the patients underwent liver transplantation (LT) with 96% and 54.5% 1-year and 5-year survival, respectively [2].

Rodriguez et al. reviewed 110 patients diagnosed with HEHE who underwent 126 transplants between 1987 and 2005 from the United UNOS database. The 1-, 3-, and 5-year patient survival rates were 80%, 68%, and 64%, respectively; 32% of those who died, died due to HEHE recurrence [3]. Lerut and his colleagues analyzed fifty-nine patients reported in the European Liver Transplant Registry; the 1-, 5-, and 10-year patient survival rates were 93%, 83%, and 72%. Nine (15.3%) patients died due to recurrent disease [4].

This patient was 40 years old at the time of transplant, similar to the average age reported by the European registry [4]. There are 6 reported cases, including ours, that received living donor liver transplantation for HEHE.

The explanted liver size was the largest reported in the literature with a value of 33 × 24 × 12 cm compared to 17 × 14 × 13 cm with the explanted liver weight of 3222 gm compared to 1250 gm. The large tumor burden may have contributed to the very early aggressive recurrence. The average time of recurrence is 49 months (6–98) but can occur up to 12 years after LT [5]. To our knowledge, this is the shortest recurrence after liver transplantation (within three months after LT).

## 3. Conclusion

Recurrence after LT in HEHE must be explored and assessed further. Survival rates at more than 10 years must also be documented and reported in the literature, as recurrence is an unpredictable event after LT.

## References

[1] Ishak K. G., Sesterhenn I. A., Goodman M. Z. D., Rabin L., Stromeyer F. W. (1984). Epithelioid hemangioendothelioma of the liver: a clinicopathologic and follow-up study of 32 cases. *Human Pathology*.

[2] Mehrabi A., Kashfi A., Fonouni H. (2006). Primary malignant hepatic epithelioid hemangioendothelioma: a comprehensive review of the literature with emphasis on the surgical therapy. *Cancer*.

[3] Rodriguez J. A., Becker N. S., O'Mahony C. A., Goss J. A., Aloia T. A. (2008). Long-term outcomes following liver transplantation for hepatic hemangioendothelioma: the UNOS experience from 1987 to 2005. *Journal of Gastrointestinal Surgery*.

[4] Lerut J. P., Orlando G., Adam R. (2007). The place of liver transplantation in the treatment of hepatic epitheloid hemangioendothelioma: report of the European liver transplant registry. *Annals of Surgery*.

[5] Rude M. K., Watson R., Crippin J. S. (2014). Recurrent hepatic epithelioid hemangioendothelioma after orthotopic liver transplantation. *Hepatology*.

